# Host factor *RBMX2* promotes epithelial cell apoptosis by downregulating APAF-1’s Retention Intron after *Mycobacterium bovis* infection

**DOI:** 10.3389/fimmu.2024.1431207

**Published:** 2024-09-06

**Authors:** Chao Wang, Yanzhu Jiang, Zhiming Yang, Haojun Xu, Abdul Karim Khalid, Tahira Iftakhar, Yongchong Peng, Lu Lu, Lei Zhang, Luiz Bermudez, Aizhen Guo, Yingyu Chen

**Affiliations:** ^1^ The National Key Laboratory of Agricultural Microbiology, College of Veterinary Medicine, Huazhong Agricultural University, Wuhan, China; ^2^ National Animal Tuberculosis Para-Reference Laboratory (Wuhan) of Ministry of Agriculture and Rural Affairs, Huazhong Agricultural University, Wuhan, China; ^3^ Department of Biomedical Sciences, College of Veterinary Medicine, Oregon State University, Corvallis, OR, United States; ^4^ Hubei Hongshan Laboratory, Huazhong Agricultural University, Wuhan, China

**Keywords:** RBMX2, *Mycobacterium tuberculosis* variant bovis, embryo bovine lung cells (EBL), apoptosis, molecular docking, APAF-1, RNA alternative splicing

## Abstract

The *Mycobacterium tuberculosis* variant bovis (*M. bovis*) is a highly pathogenic environmental microorganism that causes bovine tuberculosis (bTB), a significant zoonotic disease. Currently, “test and culling” is the primary measure for controlling bTB, but it has been proven to be inadequate in animals due to their high susceptibility to the pathogen. Selective breeding for increased host resistance to bTB to reduce its prevalence is feasible. In this study, we found a vital host-dependent factor, *RBMX2*, that can potentially promote *M. bovis* infection. By knocking *RBMX2* out, we investigated its function during *M. bovis* infection. Through transcriptome sequencing and alternative splicing transcriptome sequencing, we concluded that after *M. bovis* infection, embryo bovine lung (EBL) cells were significantly enriched in RNA splicing associated with apoptosis compared with wild-type EBL cells. Through protein/molecular docking, molecular dynamics simulations, and real-time quantitative PCR, we demonstrated that *RBMX2* promotes the apoptosis of epithelial cells by upregulating and binding to apoptotic peptidase activating factor 1 (APAF-1), resulting in the alternative splicing of APAF-1 as a retention intron. To our knowledge, this is the first report of *M. bovis* affecting host epithelial cell apoptosis by hijacking *RBMX2* to promote the intron splicing of downstream APAF-1. These findings may represent a significant contribution to the development of novel TB prevention and control strategies.

## Introduction

*Mycobacterium tuberculosis* complex (MTBC) is a microorganism that has existed in the environment for thousands of years. Current research indicates that MTBC is indirectly transmitted to humans and animals in the environment, thereby affecting their health and the economics related to its prevention and control (37). In most areas of the world where tuberculosis (TB) exists, *Mycobacterium tuberculosis* variant bovis (*M. bovis*) is a member of the MTBC and the main pathogen of bovine TB (bTB), which is designated as a mandatory notifiable disease by the World Organization for Animal Health and classified as a Class II animal disease in China (1). A study demonstrated the existence of dormant MTBC in the environment and, most importantly, its ability to restore metabolic activity when growth conditions are not favorable (38). Therefore, infection with *M. bovis* from environmental sources may be a severe risk factor for human and animal health (39). In 2012, the Chinese government listed bTB as one of the most attention-seeking diseases for prevention and control in domestic animals (1). It is becoming a significant and challenging disease worldwide, including in China (1). The main control strategy for bTB globally is based on “test and culling” (1). However, it has proven to be insufficient in eradicating the disease due to the high susceptibility of animals to this pathogen and the development by *M. bovis* of advanced metabolic activities (2). The presence of bTB may be due in part to the higher number of slaughtered animals or the lower accuracy of current diagnostic methods due to high infection rates (2). Quantitative genetic studies have shown genetic variation in cattle resistance to bTB; therefore, selective breeding for increased host resistance to bTB would be a feasible way of reducing its prevalence (3).

Apoptosis, a pivotal biological process, influences the fate of pathogenic *M. bovis* and *M. tuberculosis* during host-pathogen interactions (4). It is triggered by *M. bovis* infection, facilitating bacterial antigen presentation, reducing viable bacteria, and promoting host cell death (5–7). In the process, *M. bovis* can also hijack the host’s RNA binding proteins (RBPs) and promote the alternative splicing of apoptosis-related RNAs. For example, the Rv3654c protein of *M. tuberculosis* can bind to polypyrimidine tract-binding protein-associated splicing factor (PSF) and splice it, thereby reducing the splicing of caspase-8 pre mRNA and regulating apoptosis (8). That is why identifying host RBPs and their target RNA splicing events after *M. bovis* infection may play an important role in the development of drug targets, diagnostic biomarkers, and anti-TB breeding animals.

In this study, using an embryo bovine lung (EBL) cell genome-wide gene knockout library and an *M. bovis*-infected-EBL cell model, we found the host-dependent factor *RBMX2*. Knockout of *RBMX2* in EBL cells can potentially inhibit cell death after *M. bovis* infection. Through transcriptomic sequencing, alternative splicing enrichment analysis, molecular docking, and RT-qPCR, it was revealed that *RBMX2* facilitates apoptosis in epithelial cells through the alternative splicing of APAF-1 after *M. bovis* infection. These findings provide a theoretical basis for elucidating the molecular mechanism by which MTBC infection can promote epithelial cell death and are expected to provide new targets and strategies for controlling TB.

## Results

### *RBMX2* is highly expressed and promotes cell death after *M. bovis* infection

**Figure 1:**
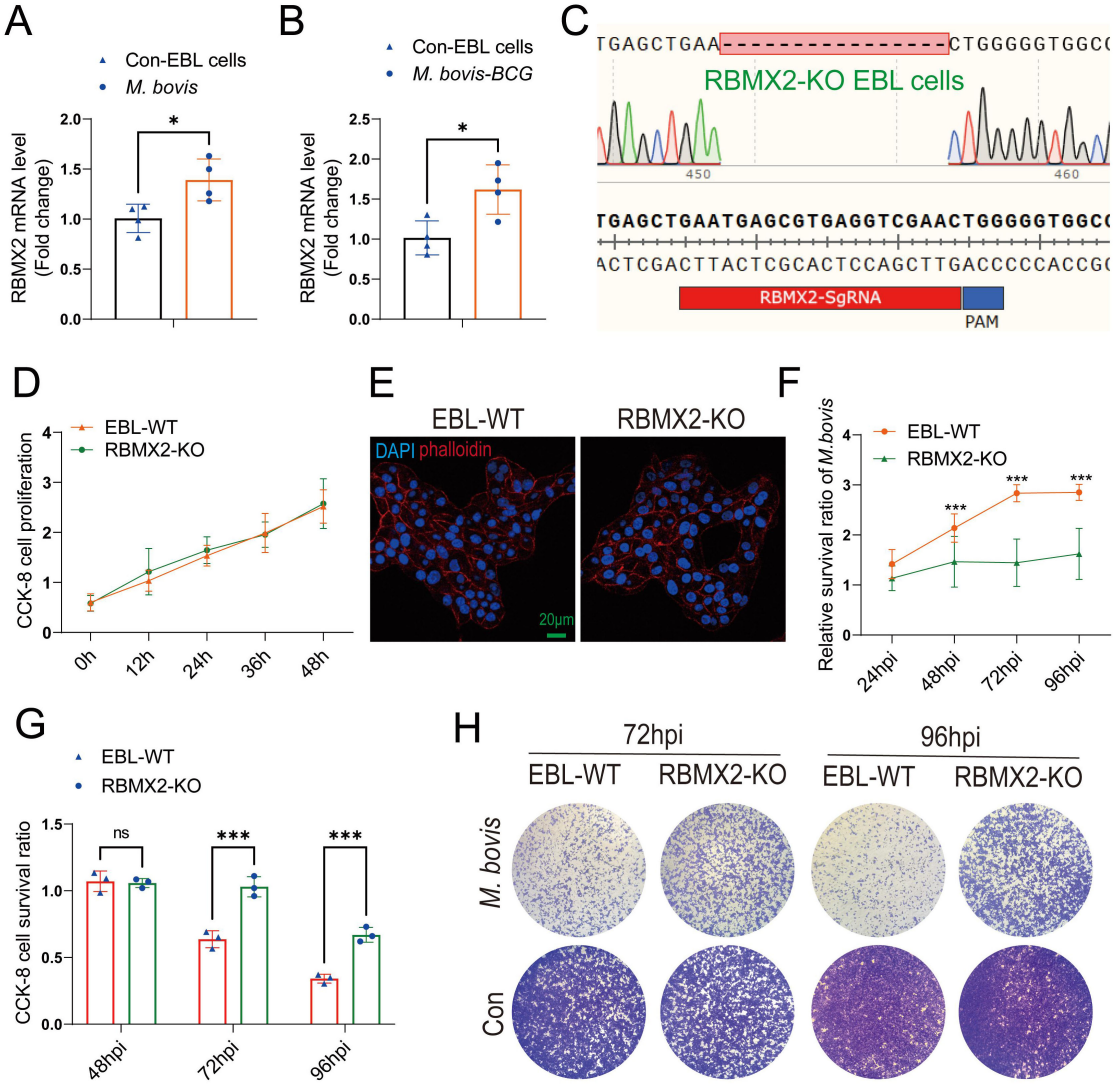
*RBMX2* was highly expressed after *M. bovis* infection and inhibited EBL cell survival. **(A, B)** The expression of *RBMX2* in EBL cells infected by **(B)** *M. bovis* (MOI, 20) and **(C)** *M. bovis-*BCG (MOI, 20) was analyzed by RT-qPCR. Data are represented as the fold expression relative to uninfected cells. **(C)** CRISPR-Cas9 technology was used to knockout *RBMX2* in EBL cells, and sequencing was used to identify 16 knocked-out bases of *RBMX2* in EBL cells. **(D)** Exploring the effect of *RBMX2* on the proliferation of EBL cells through a CCK-8 assay. Data are represented as the absorbance value relative to wild-type (WT) EBL cells. **(E)** Using phalloidine to stain the EBL cell skeleton, *RBMX2* was observed under a high-intensity microscope without affecting cell morphology. DAPI stains the cell nucleus. Scale bar: 20 μm. **(F)** The impact of *M. bovis* on the intracellular survival of *RBMX2* knocked-out EBL cells through plate counting. Data were relative to wild-type EBL cells after *M. bovis* infection. **(G, H)** *RBMX2* knockout EBL cells against *M. bovis* infection by a CCK-8 assay and Crystal Violet staining. Data are presented as the absorbance value relative to wild-type EBL cells after *M. bovis* infection (MOI 100). A t-test and two-way ANOVA were used to determine the statistical significance of differences between different groups. ns, no significance; *p < 0.05; ***p < 0.001. Data are representative of at least three independent experiments.

*RBMX2* knockout and wild-type EBL cells were infected with *M. bovis* and *M. bovis* Bacillus Calmette–Guerin (BCG), respectively, at an MOI of 20:1. RT-qPCR results indicated that the expression level of *RBMX2* was significantly upregulated after both *M. bovis* and BCG infection (**Figures 1A, B**).

To investigate the phenotype and molecular mechanism of *RBMX2* regulation in bovine lung epithelial cells infected with *M. bovis*, we used CRISPR-Cas9 technology to knockout *RBMX2* in EBL cells. The sequencing results showed that *RBMX2* knockout 16 bases (**Figure 1C**). Furthermore, a CCK-8 assay demonstrated that *RBMX2* does not affect the proliferation of EBL cells (**Figure 1D**). In addition, to investigate whether the knockout of *RBMX2* alters the morphology of EBL cells, we stained the cytoskeleton with phalloidine, and confocal microscopy results showed that *RBMX2* knockout does not alter cell morphology in EBL cells (**Figure 1E**).

A plate-counting assay was used to investigate whether *RBMX2* regulates the intracellular survival of *M. bovis* and found that *RBMX2* knockout EBL cells significantly inhibited the intracellular survival ability of *M. bovis* compared with wild-type EBL cells (**Figure 1F**). Simultaneously, a CCK-8 assay and Crystal Violet staining uncovered that after *M. bovis* infection, *RBMX2* knockout EBL cells had significantly higher CCK-8 cell proliferation levels than wild-type EBL cells at both 72 and 96 hours post-infection (hpi) (**Figures 1G, H**). These findings indicated that the mRNA expression level of *RBMX2* can be induced by *M. bovis* and promote cell death.

### RNA-seq revealed an association between *RBMX2*-regulated genes and apoptosis

**Figure 2:**
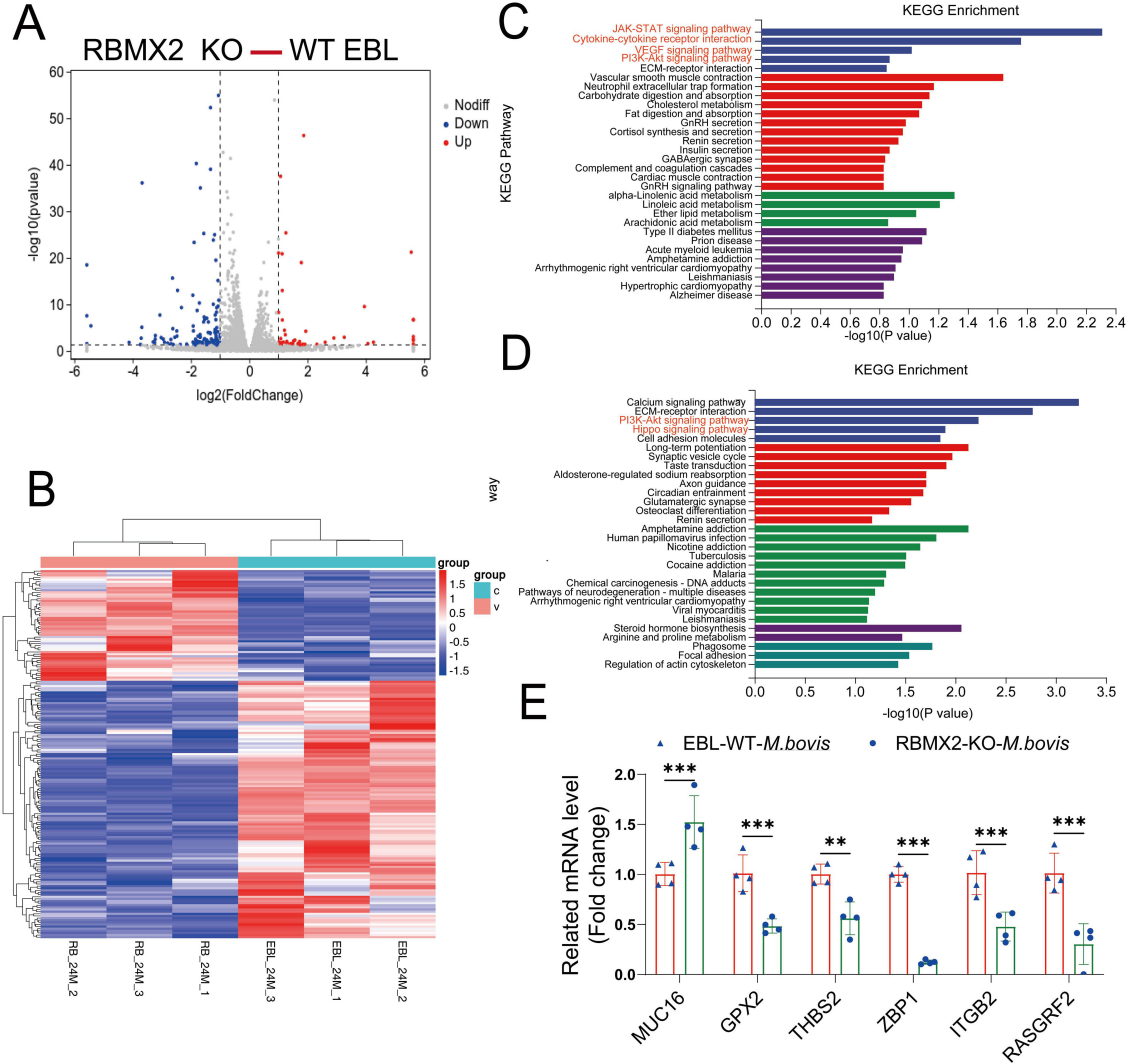
*RBMX2*-regulated genes associated with apoptosis-related pathways after infection with *M. bovis*. **(A)** The volcano plot represents differentially expressed genes in *RBMX2* knockout EBL cells and wild-type (WT) EBL cells after *M. bovis* infection (MOI, 20) 24 hpi. Red represents upregulated genes and blue represents downregulated genes. **(B)** The heat map illustrates some differentially expressed genes that were all enriched in *RBMX2* knockout EBL cells and wild-type EBL cells after *M. bovis* infection (MOI, 20) 24 hpi. Red represents upregulated genes and blue represents downregulated genes. Each group represents three independent samples. **(C, D)** KEGG Analysis of upregulated **(C)** and downregulated **(D)** genes in 24 hpi. Data representing upregulated and downregulated enriched pathways in *RBMX2* knockout EBL cells relative to WT EBL cells after *M. bovis*-infection (MOI 20). A red-marked region represents the pathways that the research focuses on. **(E)** Detecting the mRNA expression levels of RNA-seq results using RT-qPCR, including *MUC16*, *GPX2*, *THBS2*, *ZBP1*, *ITGP2*, and *RASGRF2*. Data are represented as the relative mRNA levels in *RBMX2* knockout EBL cells relative to wild-type EBL cells with *M. bovis* infection. A two-way ANOVA was used to determine the statistical significance of differences between different groups. **p < 0.01, ***p < 0.001. Data are representative of at least three independent experiments.

RNA-seq analysis was conducted to determine how *RBMX2* promotes cell death. A total of 173 genes showed significant variation in expression, with 52 upregulated and 121 downregulated in *RBMX2* knockout EBL cells compared with WT EBL cells after *M. bovis* infection (**Figures 2A, B**) (**Supplementary Table 1**). The upregulated genes were primarily related to apoptosis-related pathways such as the JAK-STAT signaling, cytokine-receptor interaction, VEGF signaling, and PI3K-Akt signaling pathways (**Figure 2C**). Conversely, the downregulated genes were primarily associated with innate immune pathways such as the PI3K-Akt signaling and Hippo signaling pathways (**Figure 2D**).

Furthermore, to verify the accuracy of transcriptome sequencing, we randomly selected six genes that regulate apoptosis. The results indicate that the transcriptome sequencing results are consistent with the RT-qPCR results (**Figure 2E**). Overall, RNA-seq revealed an association between RBMX2-regulated genes and apoptosis.

### *RBMX2* promotes apoptosis after *M. bovis* infection

**Figure 3:**
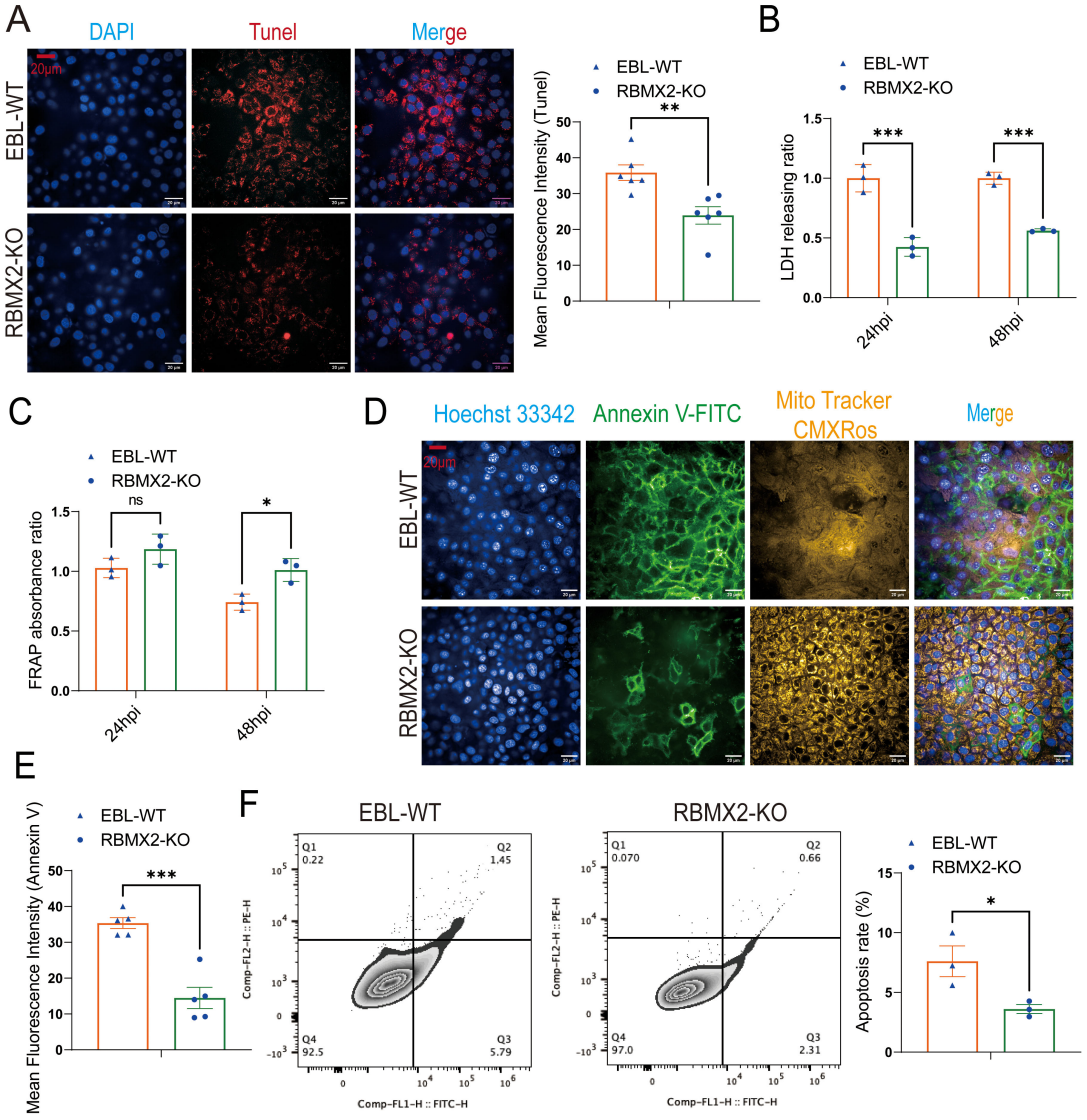
Host factor: *RBMX2* promotes apoptosis after *M. bovis* infection. **(A)** A TUNEL assay was used to detect the level of cell apoptosis. Scale bars: 20 μm. Data are presented as the red fluorescence intensity in *RBMX2* knocked-out EBL cells relative to wild-type EBL cells with *M. bovis* infection. **(B)** An LDH assay was used to detect the level of cell apoptosis. Data are presented as the LDH releasing ratio in *RBMX2* knocked-out EBL cells relative to wild-type EBL cells with *M. bovis* infection. **(C)** A FRAP assay was used to detect the level of antioxidant capacity. Data are presented as the antioxidant capacity in *RBMX2* knockout EBL cells relative to wild-type EBL cells with *M. bovis* infection. **(D, E)** A high-content imaging assay was used to detect the level of Annexin V-FITC and ROS. Scale Bar: 20μm. Data are presented as the level of mitochondrial membrane potential and ROS relative in *RBMX2* knockout EBL cells to wild-type EBL cells with *M. bovis* infection. **(F)** Flow cytometry was used to detect the level of Annexin V. Data are presented as the level of the apoptosis rate relative in *RBMX2* knockout EBL cells to wild-type EBL cells with *M. bovis* infection. A t-test and two-way ANOVA were used to determine the statistical significance of differences between different groups. ns, no significance, *p < 0.05; **p < 0.01; ***p < 0.001. Data are representative of at least three independent experiments.

Based on the transcriptome sequencing results, we detected the cell apoptosis level and lactate dehydrogenase (LDH) release in *RBMX2* knockout EBL cells and wild-type EBL cells after infection with *M. bovis*. Under confocal imaging, apoptotic cells were stained red via a terminal deoxynucleotidyl transferase-mediated dUTP nick-end labeling assay (TUNEL). There was a stronger red fluorescence in wild-type EBL cells than in *RBMX2* knockout EBL cells (**Figure 3A**). In addition, there was a significant decrease in the release of LDH in *RBMX2* knockout EBL cells after infection with *M. bovis* compared with wild-type EBL cells via an LDH assay (**Figure 3B**), which meant *RBMX2* promotes a high level of apoptosis after *M. Bovis* infection.

A ferric ion-reducing antioxidant power (FRAP) experiment was conducted to determine whether *RBMX2* increases apoptosis by modulating the host’s antioxidant stress capacity following *M. bovis* infection. Findings showed that *RBMX2* knockout EBL cells had a prominent promotion in antioxidant capacity compared with wild-type EBL cells after infection with *M. bovis* (**Figure 3C**).

*M. bovis* BCG is the attenuated strain of *M. bovis*, and now it is the only official licensed vaccine for human TB. Therefore, we need to find more antigenic markers for controlling TB. To determine whether different virulent strains of *M. bovis* have different effects on the regulation of apoptosis by *RBMX2*, we infected *RBMX2* knockout EBL cells and wild-type EBL cells with *M. bovis* BCG and detected the changes in Annexin V-FITC and CMXRos through high-intensity microscopy and flow cytometry. Under the high-content confocal cell imaging analysis system, the membrane of apoptotic cells was stained bright green by Annexin V-FITC, while normal mitochondria were stained bright yellow by Mito Tracker CMXRos. Based on the high mitochondrial membrane potential in normal cells, the results showed green solid and obscure faint yellow signals in wild-type EBL cells, which uncovered that *RBMX2* can promote apoptosis after infection with both virulent *M. bovis* and avirulent *M. bovis* BCG (**Figures 3D–F**).

### *RBMX2* promotes apoptosis through the caspase-3 pathway after *M. bovis* infection

**Figure 4:**
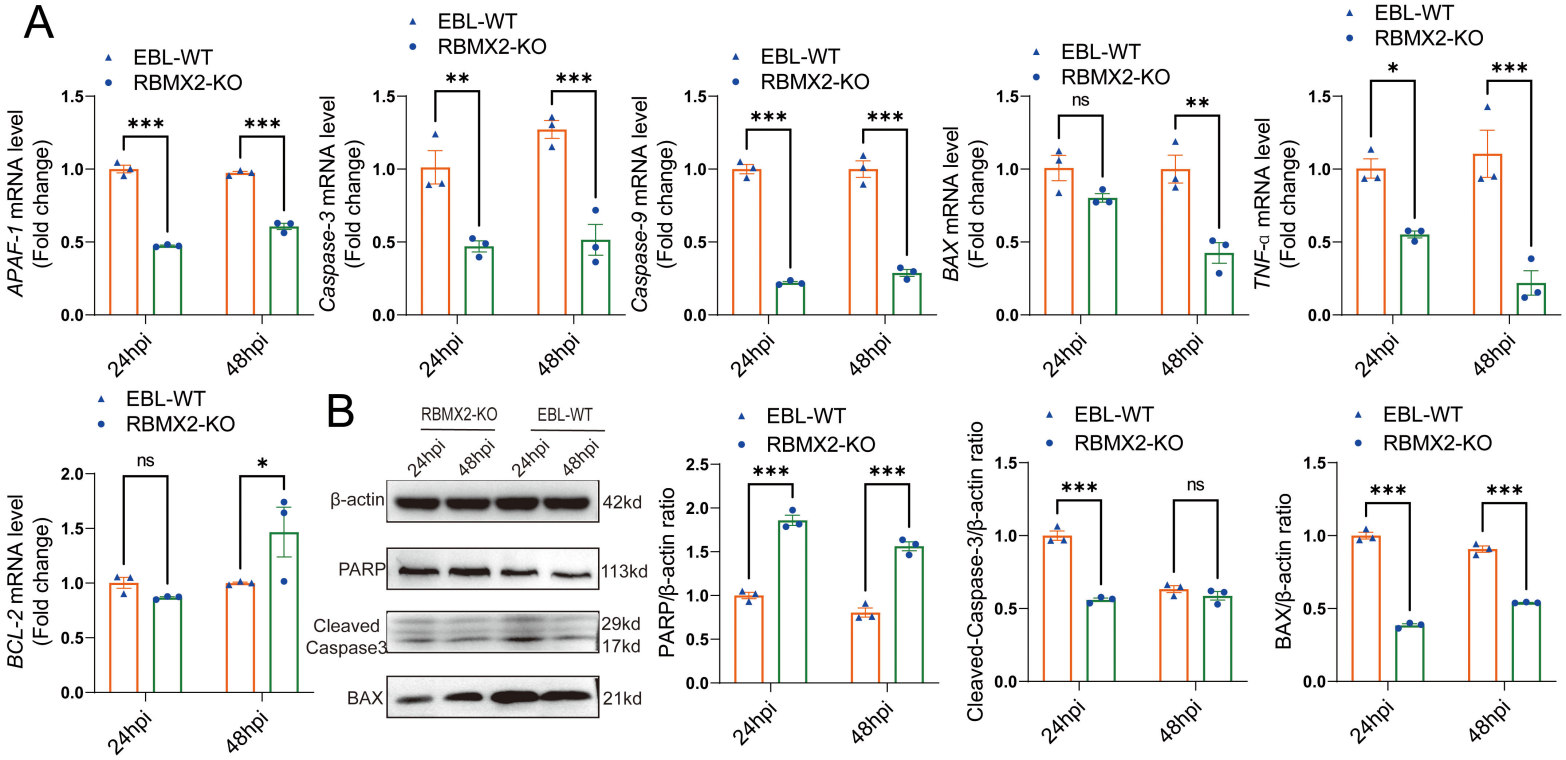
Host factor *RBMX2* promotes apoptosis through the caspase-3 pathway after *M. bovis* infection. **(A)** Detecting the mRNA expression levels of key apoptotic regulators using RT-qPCR, including BAX, APAF-1, TNF-a, caspase-9, caspase-3, and BCL-2. Data are represented as the relative mRNA level in *RBMX2* knockout EBL cells relative to wild-type (WT) EBL cells with *M. bovis* infection. **(B)** Assessing the protein expression levels of key apoptotic regulators using western blotting, including BAX, PARP, and cleaved-caspase-3. Data are presented as the relative protein level in *RBMX2* knocked-out EBL cells relative to wild-type EBL cells with *M. bovis* infection. Two-way ANOVA was used to determine the statistical significance of differences between different groups. ns, no significance, *p < 0.05; **p < 0.01; ***p < 0.001. Data are representative of at least three independent experiments.

The above comprehensive assays confirmed that *M. bovis* infection can promote *RBMX2* upregulation and induce EBL cell apoptosis. To determine the specific mechanism, we examined the levels of apoptosis-related genes (*BAX*, *APAF-1*, *TNF-α*, *Caspase-9*, *Caspase-3*, and *BCL-2*) in EBL cells following infection with *M. bovis*. RT-PCR results showed that *RBMX2* knockout cells exhibited a significant downregulation of pro-apoptotic genes (*BAX*, *APAF-1*, *TNF-a*, *Caspase-9*, and *Caspase-3*), whereas *BCL-2* was notably upregulated (**Figure 4A**). Furthermore, *RBMX2* knockout EBL cells exhibited reduced expression of pro-apoptotic proteins (BAX and cleaved-caspase-3) and an increase in PARP. Collectively, our results underscore the significance of *RBMX2* in regulating the apoptotic response to *M. bovis* infection; its knockout led to the suppression of apoptosis through the inhibition of caspase-3 cleavage (**Figure 4B**).

### *RBMX2* regulates RNA alternative splicing related to apoptosis after *M. bovis* infection

**Figure 5:**
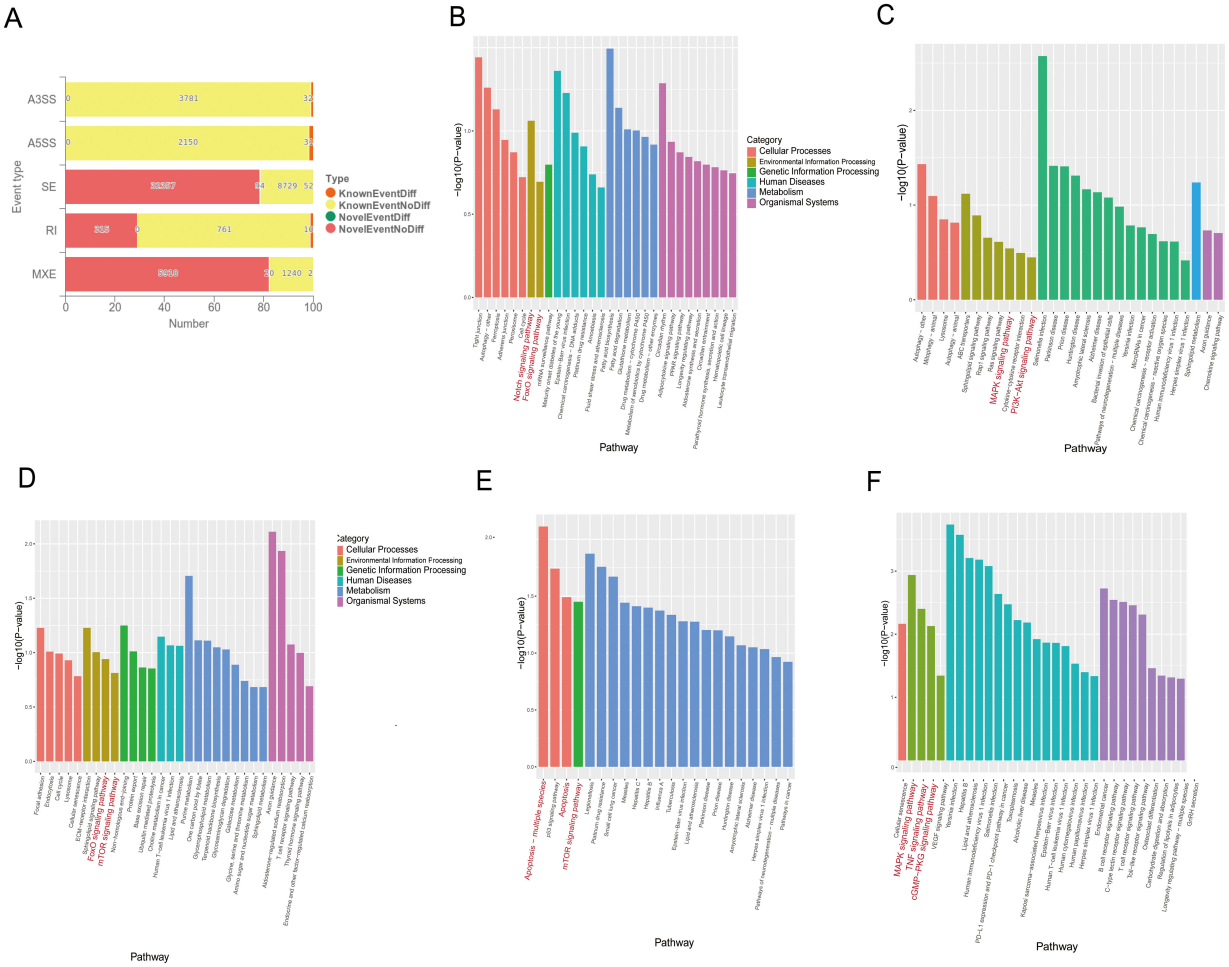
*RBMX2* regulates RNA alternative splicing associated with apoptosis after *M. bovis* infection. **(A)** Number of different RNAs with five different alternative splicing events occurring after knockout *RBMX2* in EBL cells compared with wild-type (WT) EBL cells after *M. bovis* infection. **(B–F)** KEGG analysis of RNAs involved in events such as 3′ **(B)** and 5′ splicing **(C)**, exon skipping **(D)**, intron retention **(E)**, and mutually exclusive exons **(F)** in *RBMX2* knockout EBL cells relative to wild-type EBL cells with *M. bovis* infection.

*M. tuberculosis* and its bovine *variant* strain *M. bovis* are specialized intracellular bacteria that have evolved complex and sophisticated strategies to evade host surveillance systems (9, 10). It has been found that *M. tuberculosis* and *M. bovis* can influence host cell-RBP-mediated RNAs by alternative splicing related to apoptosis. *RBMX2*, as an RBP, plays an important role in alternative splicing. However, it is still unknown which RNAs are enriched by alternative splicing to promote apoptosis after *M. bovis* infection. Through transcriptome sequence analysis, we found that 241 RNAs were involved in five different alternative splicing patterns in *RBMX2* knockout EBL cells compared with wild-type EBL cells, among which 63 RNAs undergo variable 3’ (A3SS) and 5’ splicing (A5SS), 146 RNAs undergo exon skipping (SE), 10 RNAs undergo intron retention (RI), and 22 RNAs undergo mutually exclusive exon (MXE) splicing (**Figure 5A**). KEGG pathway analysis revealed that the A3SS-related RNAs were related to the Notch signaling and FoxO signaling pathways (**Figure 5B**). A5SS-related RNAs were linked to the MAPK signaling and PI3K-Akt signaling pathways (**Figure 5C**), and RNAs involved in SE were annotated in the FoxO signaling and mTOR signaling pathways (**Figure 5D**). RI-related RNAs were annotated in the apoptosis and the mTOR signaling pathways (**Figure 5E**), and the MXE-related RNAs were annotated in the MAPK signaling, TNF signaling, and cGMP-PKG signaling pathways (**Figure 5F**). As all the pathways mentioned above are potentially related to apoptosis, it was speculated that *RBMX2* induces apoptosis through the promotion of alternative splicing after *M. bovis* infection.

### *RBMX2* promotes apoptosis by regulating the alternative splicing of APAF-1’s RI variant

**Figure 6:**
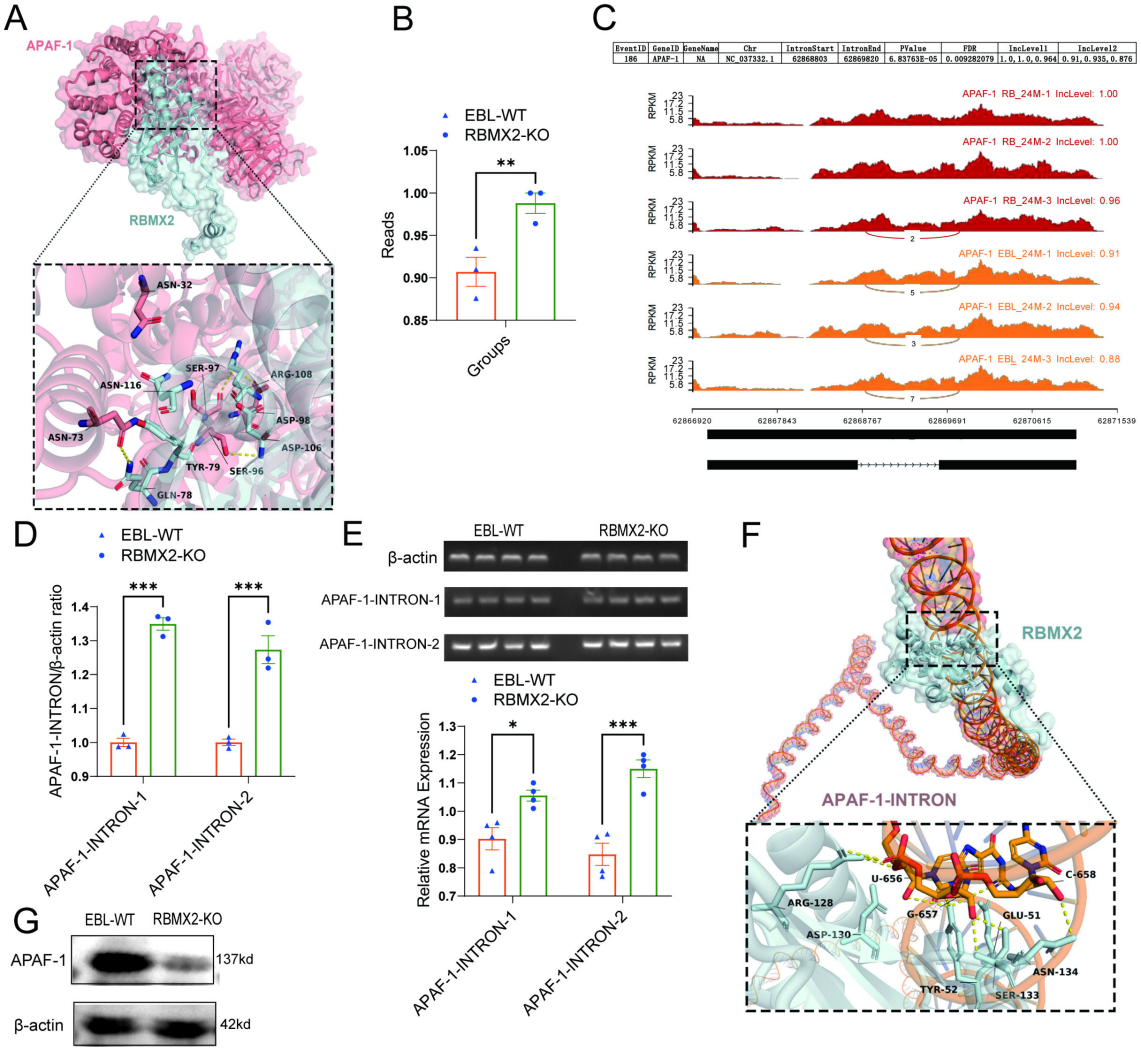
*RBMX2* promotes apoptosis in epithelial cells by regulating the alternative splicing of APAF-1’s RI isoform. **(A)** Conformational diagram of binding sites between *RBMX2* protein and APAF-1 protein. **(B)** The reads of retaining introns in AFAP1 in *RBMX2* knockout EBL cells compared with wild-type (WT) EBL cells after *M. bovis* infection. **(C)** Sequencing APAF-1 RNA in *RBMX2* knocked-out EBL cells compared with wild-type EBL cells after *M. bovis* infection and visualizing its splicing patterns using software such as rMATS. **(D, E)** Design of two pairs of primers targeting the intron region (62868803-62869820) of APAF-1 RNA and using RT-qPCR to detect this intron region in *RBMX2* knockout EBL cells and wild-type EBL cells after *M. bovis* infection. **(F)** The conformational diagram of the binding site between *RBMX2* protein and APAF-1 retained introns. **(G)** Detecting the protein expression of APAF-1 using a western blot assay. Data are presented as the relative protein level in *RBMX2* knockout EBL cells relative to wild-type EBL cells with *M. bovis* infection. A t-test and two-way ANOVA were used to determine the statistical significance of differences between different groups. *p < 0.05; **p < 0.01; ***p < 0.001. Data are representative of at least three independent experiments.

APAF-1 is a core apoptotic factor that promotes apoptosis through apoptosome formation and by activating caspase-3 (11). RNA-seq shows that APAF-1 is listed among the 241 alternative splicing-related RNAs. Through protein-protein docking, we found that the 73 ASN and 96 SER sites of APAF-1 tightly bind to the 78 GLN and 106 ASP sites of *RBMX2* (**Figure 6A**).

By using rMATS software, APAF-1 (NC_037332.1) presented a higher RI within the region 62868803-62869820 in *RBMX2* knockout EBL cells than in wild-type EBL cells (**Figures 6B, C**). To verify the bioinformatic results, we employed an RT-qPCR assay to detect the intron region of APAF-1. The results indicated that *RBMX2* knockout EBL cells and significantly increased the expression of the 62868803-62869820 intronic sequence compared with wild-type EBL cells (**Figures 6D, E**). By using the protein-RNA docking technique, we predicted the structural interaction between *RBMX2* protein and APAF-1 RNA. We identified multiple spatial sites in the *RBMX2* protein and APAF-1 intron regions, such as 656U, 657G, and 658C (**Figure 6F**). In *RBMX2* knockout EBL cells, APAF-1 was significantly decreased after *M. bovis* infection (**Figure 6G**). Along with it, *RBMX2* can promote epithelial cell apoptosis after *M. bovis* infection by regulating the alternative splicing of the apoptosis-related RNA of APAF-1. 

## Discussion

bTB, which is primarily caused by *Mycobacterium bovis* (12), poses a significant threat to both animal and human health and has serious implications for the environment, agriculture, and economic development worldwide (13). In domestic and wild animals, the transmission of *M. bovis* may occur through aerosols, the inhalation of infectious droplets, or infected body fluids or tissues (40). *M. bovis* serves as an important environmental pathogenic microorganism (41); the failure of “test and culling” in many countries, including China, highlights the need for more targeted prevention and control strategies (14).

Host-dependent factors with anti/resistant disease ability play a crucial role in the control of *M. bovis*. In domestic and wild animals, selective breeding with resistant genes against different pathogens may prove to be a sustainable way of controlling infection. (15, 16). These host-dependent factors can be regulated through genetic improvement and gene editing, contributing to the breeding of animals with higher resistance to specific infectious diseases (17, 18). A previous study reported that double knockout of host-dependent factors CD163 and pAPN in pigs confers resistance to PRRSV and TGEV (19). Transcription activator-like effector (TALE) nickase-mediated SP110 knockout bestows cattle a higher resistance to TB (20). Therefore, the exploration and use of *M. bovis* host-dependent factors provide crucial theoretical and technical support for TB-resistant breeding, leading to the breeding of animal varieties with increased TB resistance and improving the health and economic benefits of the livestock industry.

*RBMX2*, an RNA-binding motif, plays an important role in mRNA splicing via spliceosomes; data from past studies showed that it is regarded as an important molecular marker for assessing sperm activity (21). *RBMX2* also serves as an X-chromosome-specific gene in telomeric cells (42).

In this study, we found that *RBMX2* was increased after *M. bovis* infection and can lead to the apoptosis of infected EBL cells. To our knowledge, this is the first investigation that reported and highlighted the function of *RBMX2* in *M. bovis* infection.

Apoptosis, or programmed cell death, is a fundamental biological process that plays a dual role in host defense against pathogens (22–24). Apoptosis facilitates the elimination of intracellular pathogens by promoting their engulfment and phagocyte degradation (25). *M. bovis*/*M. tuberculosis* infection may promote host cell apoptosis through various mechanisms, such as disrupting the intracellular environment and activating apoptosis-related pathways (26). *M. bovis* infection can lead to inflammation and tissue damage, increase the activity of immune cells, and promote apoptosis to combat the infection (26–28). Here, we found that *RBMX2* was increased after *M. bovis* infection, leading to significant apoptosis. This indicated that *M. bovis* induced host cells to express more *RBMX2* to induce cell death, through which they combat the infection.

RNA alternative splicing (AS), mediated by RBPs, is a highly regulated process that plays a pivotal role in cellular homeostasis and stress response (29). RBPs can modulate gene expression by regulating AS patterns, thereby influencing diverse biological processes (30). Very few studies have reported how bacteria utilize host RBPs to regulate the alternative splicing (AS) of target RNAs to promote infection and pathogenicity. Limited studies were found to mainly focus on *Escherichia coli* and *Helicobacter pylori* infection (31, 32), and reported the enrichment of phosphorylated proteomic RNA and alternative splicing after *H. pylori* infection in gastric cells (33). However, how *M. tuberculosis*/*M. bovis* utilizes host RBPs to regulate the AS of target RNAs to promote infection and pathogenicity has rarely been reported so far (34, 35). In this study, *RBMX2* was proven to promote apoptosis through AS and was especially involved with APAF-1, a critical regulator of apoptosis (36).

Furthermore, we observed higher RI within the 62868803-62869820 region of the APAF-1 (NC_037332.1) transcript in *RBMX2* knockout EBL cells than in WT EBL cells after *M. bovis* infection, suggesting a potential molecular mechanism by which *RBMX2* modulates to apoptosis through APAF-1 splicing. Protein-protein docking and molecular dynamics simulations further supported the interaction between *RBMX2* and APAF-1, highlighting the functional relevance of their association in regulating apoptosis. However, the precise mechanism through which *RBMX2* modulates APAF-1 splicing and its impact on *M. bovis* pathogenesis must be further investigated. However, owing to the fact that *M. bovis* is a high-grade pathogen, many experiments have not yet been well conducted, such as live-cell imaging and flow cytometry. Therefore, in this article, we partially used attenuated BCG to infect bovine lung epithelial cells to explore the mechanism of RBMX2-mediated apoptosis. In addition, RBMX2, as a global splicing regulatory factor, plays an important role in the host infection of *M. bovis*. In this article, we only focus on studying and discussing its related mechanisms for mediating apoptosis; other enriched pathways and downstream factors will be the focus of our future work.

In summary, we identified *RBMX2* as a key regulator of apoptosis after *M. bovis* infection and systematically validated that *M. bovis* hijacks the upregulation of *RBMX2* expression and promotes the cleavage of APAF-1 introns, increasing its translation, and then regulates host epithelial cell apoptosis after *M. bovis* infection. Our findings may provide a novel insight into the molecular mechanisms underlying host-pathogen interactions during *M. bovis* infection and may point to a new approach to bTB control through selective breeding.

## Conclusion

**Figure 7:**
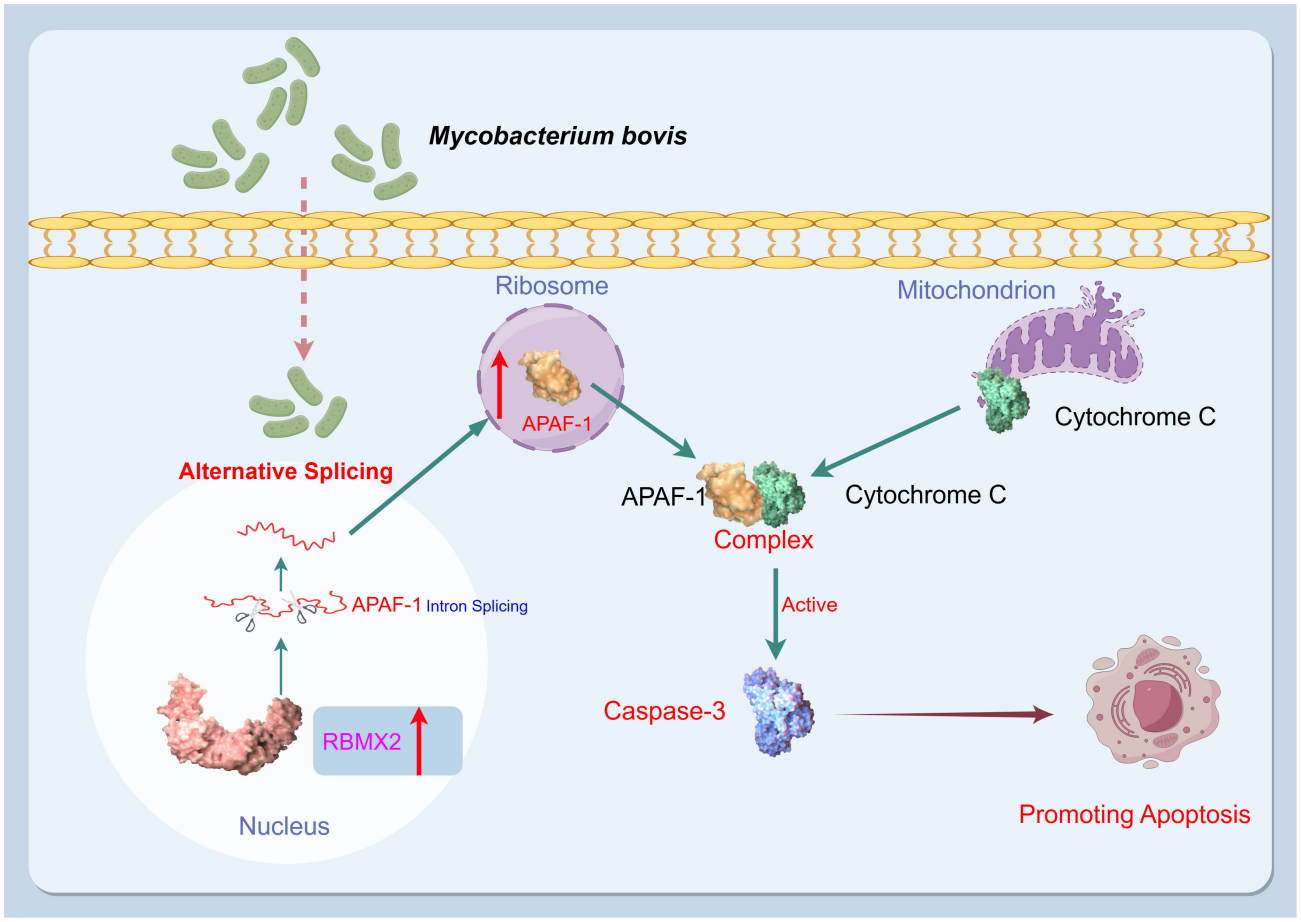
Mechanism diagram. After infection with *M. bovis*, *RBMX2* is upregulated in EBL cells. *RBMX2* then increases the translation of APAF-1 RNA and expression of APAF-1 protein by downregulating its retained intron, thereby improving its binding to cytochrome C. Finally, the APAF-1 and cytochrome C complexes activate caspase-3 to promote apoptosis in epithelial cells following *M. bovis* infection.

This study identified a novel host factor, *RBMX2*, implicated in the promotion of cell death after *M. bovis* infection and the facilitation of apoptosis in epithelial cells through the AS of APAF-1, which activates caspase-3 by forming the apoptosome (**Figure 7**). These findings provide a new target for the prevention and control of TB based on the genomics of host anti-TB.

## Materials and methods

### Cell lines

EBL cells were kindly provided by M. Heller from the Friedrich-Loeffler Institute, Germany. These cells were grown in heat-inactivated 10% fetal bovine serum supplemented with Dulbecco’s modified Eagle medium (Gibco, USA) at 37°C and 5% CO_2_.

### Bacterium culture and infection

*M. bovis* [American Type Culture Collection (ATCC): 19210] was kindly provided by Dr. Chuanyou Li (Beijing Tuberculosis and Thoracic Tumor Research Institute, Beijing, China). *M. bovis* BCG-Pasteur (ATCC: 35734) was a gift from Professor Luiz Bermudez (Oregon State University). All strains were cultured in a Middlebrook 7H9 broth (BD, MD, USA) containing 0.5% glycerol (Sigma-Aldrich, MO, USA), 10% oleic acid-albumin-dextrose-catalase (OADC, BD), and 0.05% Tween 80 (Sigma-Aldrich) or on Middlebrook 7H11 agar plates (BD, MD, USA) containing 0.5% glycerol (Sigma-Aldrich) and 10% OADC (BD). Before infection, the optical densities at 600 nm (OD600) of the bacterial culture were adjusted to the required MOI using the standard turbidimetric method. The cultures were then centrifuged at 3,000 × g for 10 min. The precipitated bacteria were resuspended in a medium and dispersed by passage through an insulin syringe. An aliquot of 50 μl of the 10-fold serially diluted bacterial suspension was plated onto Middlebrook 7H11 agar (BD) to count viable bacteria (colony-forming units, CFUs).

For the infection, both *M. bovis* and *M. bovis* BCG were used to infect EBL cells at MOIs of 20 (for apoptosis detection) or 100 (for cell viability).

All *M. bovis*-related experiments in this study were conducted strictly in accordance with the biosafety-related operating procedures at the Animal Biosafety Level 3 Laboratory of the National Key Laboratory of Agricultural Microbiology at Huazhong Agricultural University.

### Extraction of total RNA and RT-qPCR

Cold phosphate-buffered saline (PBS, HyClone, China) was used to wash the cells three times, and 1 ml of Trizol (Invitrogen, USA) was added per well. The cells were lysed and collected in Eppendorf tubes. In addition, 200 μl of chloroform was added, followed by vortexing for 30 s and centrifugation at 12,000 rpm at 4°C for 10 min. Afterward, 500 μl of the supernatant was collected in a new Eppendorf tube. Then, 500 μl of isopropanol was added to the collected supernatant, followed by gradually mixing upside down. The mixture was allowed to stand for 10 min at 4°C, followed by centrifugation at 12,000 rpm at 4°C for 15 min. The supernatant was removed, and the RNA pellet was visible. Next, 1 ml of 75% ethanol was added to wash the RNA, followed by centrifugation at 7,500 rpm at 4°C for 5 min. The supernatant was removed, and the RNA was dried for 15 min. Subsequently, 20 μl of DEPC water was added, and the mixture was incubated at 58°C in a water bath for 10 min. Finally, purified RNA was obtained.

As a quality control index of RNA purity, OD260/OD280 values between 1.8 and 2.0 were determined using a NanoDrop ND-1000 instrument (Agilent Inc., USA). Denaturing agarose gel electrophoresis was used to measure RNA integrity and contamination with gDNA. The sample was stored at −80°C for further analysis.

Reverse transcription of the RNA samples was performed using HiScript III RT SuperMix for qPCR (+gDNA wiper, Vazyme, China). A volume of 4 μl of 4 × gDNA wiper mix was added to 1 μg of RNA, which was then added to 16 μl of RNAse-free ddH_2_O. The mixture was incubated at 42°C for 2 min to remove genomic DNA. Reverse transcription was performed by adding 4 μl of 5 × HiScript III qRT SuperMix. The mixture was incubated at 37°C for 15 min and then at 85°C for 5 s to obtain cDNA.

cDNA expression in different groups of samples was detected using AceQ qPCR SYBR Green Master Mix (Vazyme, China) in a ViiA7 real-time PCR machine (Applied Biosystems Inc, USA). The final volume of the real-time PCR reaction was 20 μl, including 10 μl of 2 × AceQ qPCR SYBR Green Master Mix, 0.4 μl of upstream primer (10 μM), 0.4 μl of downstream primer (10 μM), 0.4 μL of 50 × ROX reference dye 2, 3 μl of cDNA template, and 5.8 μl of ddH_2_O. The reaction was completed as follows: 95°C for 5 min (1 cycle), 95°C for 10 s (40 cycles), and 60°C for 30 s (40 cycles). **Supplementary Table 1** shows the RT-qPCR primer sequences.

### Western blot

Radioimmunoprecipitation assay reagent (Sigma-Aldrich, USA) containing protease inhibitors and phosphatase inhibitors (Roche, China) was added to the cell samples, and the cells were lysed for 30 min on ice. The supernatant was collected by centrifugation at 12,000 rpm at 4°C for 10 min. A bicinchoninic acid kit (Beyotime, China) was used to detect the protein concentration of each sample, and the proteins were adjusted to equal concentrations. The protein samples were mixed with 5 × protein loading buffer and boiled for 10 min. Proteins were separated using SDS-PAGE on 10% polyacrylamide gels at 100 V for 90 min and then transferred to polyvinylidene difluoride (PVDF) membranes (Millipore, Germany) at 100 V for 70 min. The PVDF membrane was placed in 5% skimmed milk and sealed for 4 h. The membrane was washed three times with Tris-buffered saline with 0.15% Tween-20 for 5 min. Then, the membrane was incubated with a primary antibody [cleaved-caspase3 (Bioss, China), PARP (Proteintech, China), BAX (Proteintech, China), APAF-1 (Bioss, China), and β-actin (Bioss, China)] at 4°C for 12h followed by a secondary antibody [anti-mouse HRP secondary antibody and anti-rabbit HRP (Invitrogen, USA)] at room temperature for 1 h.

### Generation of the *RBMX2*-KO EBL cells

The small guide RNA (sgRNA) sequence targeting the bovine *RBMX2* gene (5′- GAATTGCGGGGTCGAAC-3′) was cloned into pKLV2-U6gRNA5 (BbsI)-PGKpuro2ABFP (# 67991) (gifted by Professor Shuhong Zhao of Huazhong Agricultural University), and a recombinant lentivirus was constructed using a three plasmid helper system (PMD2. G and psPAX2 helper plasmids gifted by Professor Shuhong Zhao of Huazhong Agricultural University). EBL cells were infected with the *RBMX2* pKLV2−U6gRNA5(BbsI)−PGKpuro2ABFP (#67991) lentivirus or the empty vector pKLV2−U6gRNA5(BbsI)−PGKpuro2ABFP (#67991) lentivirus (negative control). After 48 h of infection, puromycin (2.0 mg/ml) was added to select positive clone cells. Finally, the monoclonal knockout cells obtained through limited dilution were amplified, and the knockout sites were identified using Sanger sequencing (**Supplementary Table S2**).

### Assay of intracellular bacterial survival

EBL cells were infected with *M. bovis* at 37°C with a MOI of 20 for 2 h. Then, the cells were washed three times with sterile 1×PBS to remove extracellular bacteria. This time point is considered as 0 h; cells in a complete culture medium containing 100 µg/ml of gentamicin were cultivated for different durations (24 h, 48 h, 72 h, and 96 h). Afterward, the cell lysate was continuously diluted and spread onto a 7H11 agar plate supplemented with 10% OADC, and CFUs were calculated after 3 weeks of cultivation. Each sample was plated three times.

### Alternative splicing RNA-Seq

Total RNA was isolated using Trizol Reagent (Invitrogen Life Technologies), and the concentration, quality, and integrity were determined using a NanoDrop spectrophotometer (Thermo Scientific). Three micrograms of RNA were used as input material for RNA sample preparation. Sequencing libraries were generated according to the following steps. mRNA was purified from total RNA by using poly-T oligo-attached magnetic beads. Fragmentation was performed using divalent cations under elevated temperature in an Illumina proprietary fragmentation buffer. First-strand cDNA was synthesized using random oligonucleotides and Super Script II. Second-strand cDNA synthesis was subsequently performed using DNA Polymerase-I and RNase H. The remaining overhangs were converted into blunt ends via exonuclease/polymerase activities, and the enzymes were removed. Illumina PE-adapted oligonucleotides were ligated for hybridization after adenylation of the 3′ ends of the DNA fragments. To select cDNA fragments of the preferred length of 400–500 bp, the library fragments were purified using the AMPure XP system (Beckman Coulter, Beverly, CA, USA). DNA fragments with ligated adaptor molecules at both ends were selectively enriched using the Illumina PCR Primer Cocktail in a 15-cycle PCR reaction. The products were purified (AMPure XP system) and quantified using the Agilent high-sensitivity DNA assay on a Bioanalyzer 2100 system (Agilent Inc., USA). The sequencing library was then sequenced on the NovaSeq 6000 platform (Illumina) by Shanghai Personal Biotechnology Cp. Ltd.

The RNA Sequencing service was provided by Personal Biotechnology Co., Ltd., Shanghai, China. The data were analyzed using the free online platform Personalbio GenesCloud (https://www.genescloud.cn). Furthermore, we randomly selected genes related to apoptosis for validation. **Supplementary Table S2** shows the RT-qPCR primer sequences.

### TUNEL assay

The TUNEL assay was performed to detect apoptotic cells after infection. Cell samples were prepared and subjected to deoxyribonucleotide triphosphates (dNTPs) blocking to minimize the background signals. The TUNEL reaction mixture, consisting of terminal deoxynucleotidyl transferase (TdT) and labeled deoxyuridine triphosphate (dUTP), was applied to the samples and incubated under humid conditions for 30 min to 1 h. Subsequently, samples were washed with PBS to remove unincorporated labeled dUTP. Optionally, selective nuclear staining was performed to visualize nuclear morphology. Finally, samples were observed using a fluorescence microscope to detect TUNEL-positive cells. Experimental procedures were carried out strictly according to the manufacturer’s instructions provided with the TUNEL assay kit (Beyotime, China).

### Apoptosis detection assay via a confocal high-content cell imaging system

After infection, a confocal high-content cell imaging analysis system was used to determine the mitochondrial membrane potential according to the instructions provided with the mitochondrial membrane potential and apoptosis detection kit (Beyotime, China). Cells were analyzed using a confocal high-content cell imaging analysis system (PerkinElmer Life And Analytical Sciences Ltd, UK).

### Apoptosis detection assay using a flow cytometer

Cold PBS was used to wash the cell samples three times after infection. Cells were digested by trypsin, and the digestion was terminated with DME/F-12 medium containing 10% FBS. Cells were collected by centrifugation at 1,500 rpm for 5 min. The samples were washed three times in PBS by centrifugation at 1,500 rpm for 5 min. The supernatant was discarded. An Annexin V-FITC Apoptosis Detection Kit (Beyotime, China) was used to detect apoptosis. An aliquot of 100 μl of binding buffer was added to resuspend the cell. EBL cells were stained with Annexin V-FITC and propidium iodide for 10 min before being detected by CytoFLEX-LX (Becton Dickinson, USA).

### LDH assay

LDH activity was determined using a commercially available assay kit (Beyotime, China). Cell samples were homogenized in ice-cold lysis buffer after infection, followed by centrifugation to collect the supernatant. LDH activity was measured by monitoring the decrease in absorbance at 340 nm over time, reflecting the rate of NADH oxidation. Protein concentrations were quantified using the Bradford assay, and LDH activity was normalized to protein content.

### Cell viability assay

EBL cells were infected with *M. bovis* at an MOI of 100. Cell viability was then detected at different hours post-infection using CCK-8 (Dojindo, Kumamoto, Japan). Briefly, at 48, 72, and 96 hpi, fresh medium containing 10% CCK-8 was added to a 96-well cell plate and incubated for 60 min at 37°C. The cell viability was then determined as the absorbance at 450 nm.

### Protein-protein docking assay

We obtained the protein structures of APAF-1 and *RBMX2* from the UniProt database (https://www.uniprot.org/) and evaluated the interaction pattern between APAF-1 and *RBMX2* using Z-dock. Pymol 2.3.0 (https://pymol.org/) was used to analyze the interaction mode of the docking results.

### Bioinformatics analysis of *RBMX2* protein and APAF-1 RNA interaction

Sequence Retrieval and Alignment: the amino acid sequence of the RBMX2 protein and the RNA sequences of APAF-1 were retrieved from the National Center for Biotechnology Information database (https://www.ncbi.nlm.nih.gov/). Multiple sequence alignments of RBMX2 protein sequences and APAF-1 RNA sequences from various species were performed using Clustal Omega software (https://www.ebi.ac.uk/Tools/msa/clustalo/).

Structural Prediction: the secondary structure of APAF-1 RNA was predicted using the RNAfold web server (http://rna.tbi.univie.ac.at/cgi-bin/RNAWebSuite/RNAfold.cgi). The tertiary structure of the RBMX2 protein was predicted using I-TASSER (https://zhanglab.ccmb.med.umich.edu/I-TASSER/).

### Statistical analysis

Statistical analysis involved conducting all assays in triplicate and expressing the data as the mean ± standard error with three independent repetitions of all experiments. Statistical analyses were performed using GraphPad Prism 7.0, with t-tests to compare two groups and two-way ANOVA to compare multiple variables.

## Data Availability

The datasets presented in this study can be found in online repositories. The names of the repository/repositories and accession number(s) can be found below: https://www.ncbi.nlm.nih.gov/, PRJNA1105205.

## Supplementary material

Supplementary Table 1 RT-qPCR primer sequences.

Supplementary Table 2
*RBMX2* sgRNA sequence.

Supplementary Table 3 RT-qPCR primer sequences.
